# Heterologous expression and characterization of novel GH12 β-glucanase and AA10 lytic polysaccharide monooxygenase from *Streptomyces megaspores* and their synergistic action in cellulose saccharification

**DOI:** 10.1186/s13068-023-02332-0

**Published:** 2023-05-24

**Authors:** Xing Qin, Kun Yang, Jiahuan Zou, Xiaolu Wang, Tao Tu, Yuan Wang, Xiaoyun Su, Bin Yao, Huoqing Huang, Huiying Luo

**Affiliations:** State Key Laboratory of Animal Nutrition, Institute of Animal Science, Chinese Academy of Agricultural Sciences, Beijing, China

**Keywords:** β-Glucanase, Lytic polysaccharide monooxygenase, Synergy, Cellulose saccharification, *Streptomyces megaspores*

## Abstract

**Background:**

The combination of cellulase and lytic polysaccharide monooxygenase (LPMO) is known to boost enzymatic saccharification of cellulose. Although the synergy between cellulases (GH5, 6 or 7) and LPMOs (AA9) has been extensively studied, the interplay between other glycoside hydrolase and LPMO families remains poorly understood.

**Results:**

In this study, two cellulolytic enzyme-encoding genes *SmBglu12A* and *SmLpmo10A* from *Streptomyces megaspores* were identified and heterologously expressed in *Escherichia coli*. The recombinant *Sm*Bglu12A is a non-typical endo-β-1,4-glucanase that preferentially hydrolyzed β-1,3-1,4-glucans and slightly hydrolyzed β-1,4-glucans and belongs to GH12 family. The recombinant *Sm*Lpmo10A belongs to a C1-oxidizing cellulose-active LPMO that catalyzed the oxidation of phosphoric acid swollen cellulose to produce celloaldonic acids. Moreover, individual *Sm*Bglu12A and *Sm*Lpmo10A were both active on barley β-1,3-1,4-glucan, lichenan, sodium carboxymethyl cellulose, phosphoric acid swollen cellulose, as well as Avicel. Furthermore, the combination of *Sm*Bglu12A and *Sm*Lpmo10A enhanced enzymatic saccharification of phosphoric acid swollen cellulose by improving the native and oxidized cello-oligosaccharides yields.

**Conclusions:**

These results proved for the first time that the AA10 LPMO was able to boost the catalytic efficiency of GH12 glycoside hydrolases on cellulosic substrates, providing another novel combination of glycoside hydrolase and LPMO for cellulose enzymatic saccharification.

**Supplementary Information:**

The online version contains supplementary material available at 10.1186/s13068-023-02332-0.

## Background

Cellulose is the most prevalent renewable carbohydrate in nature, widely found in lignocellulosic biomass, microbes, and animals [1]. It consists of linear chains of several hundred to over ten thousand glucose molecules connected by β-1,4-glycosidic linkages [2]. These long polymer chains are arranged in a highly ordered crystalline structure, severely limiting the accessibility of microbial enzymes to the cellulose chains [3]. To efficiently utilize cellulose, cellulolytic microorganisms have evolved multiple strategies to decompose cellulose into sugar monomers, including hydrolysis and oxidation [4]. As for enzymatic hydrolysis of cellulose, three main kinds of glycoside hydrolases such as cellobiohydrolase, endoglucanase, and β-glucosidase, act synergistically to break β-1,4-glycoside linkages. With respect to the oxidation cleavage of cellulose, critical enzymes known as lytic polysaccharide monooxygenases (LPMOs) catalyze the oxidative breakage of the β-1,4-glycosidic linkages in the crystalline regions using O_2_/H_2_O_2_ and electrons [5, 6]. The interplay between glycoside hydrolases and LPMOs in the enzymatic saccharification of cellulose remains to be elucidated [7, 8].

Recently, the synergistic action between glycoside hydrolases and LPMOs on the enzymatic deconstruction of cellulose has attracted more and more attention from researchers [9–13]. Several studies have reported that supplementation of cellulases from glycoside hydrolase families 5, 6 or 7 (GH5, 6 or 7) with LPMOs from the auxiliary activity family 9 (AA9) could significantly enhance enzymatic saccharification of cellulose [14, 15]. Besides, the promoting effect of AA9 LPMOs on cellulase activity is enzyme-dependent [7, 8, 10]. Specifically, C1-oxidizing AA9 LPMOs promote catalytic activity of GH5 endoglucanases and GH6 nonreducing-end cellobiohydrolases, respectively [7, 8]. C1/C4-oxidizing AA9 LPMOs significantly improve hydrolytic efficiency of GH6 nonreducing-end cellobiohydrolases and GH7 reducing-end cellobiohydrolases [10]. Although the synergistic interaction between cellulases (GH5, 6 or 7) and LPMOs (AA9) has been extensively studied, the interplay between other glycoside hydrolase and LPMO families is still poorly understood.

*Streptomyces* species are a class of efficient cellulose degraders in nature, which produce a variety of extracellular cellulose-degrading hydrolases (GH5, 6 or 12) and oxidative enzymes (AA10) [16–18]. Herein, two adjacent cellulolytic enzyme genes *SmBglu12A* and *SmLpmo10A* were identified from the genome of *Streptomyces megaspores* based on the carbohydrate-active enzymes annotation. In this work, the two genes were successfully cloned and expressed in *Escherichia coli* strains Origami DE3 and Shuffle T7-B, respectively, after screening for multiple molecular chaperones. The purified recombinant *Sm*Bglu12A and *Sm*Lpmo10A were biochemically characterized, with particular attention to their performance in degrading PASC (phosphoric acid swollen cellulose). The synergistic action between *Sm*Bglu12A and *Sm*Lpmo10A in the enzymatic saccharification of PASC was evaluated. The results demonstrated for the first time that the combination of *Sm*Bglu12A and *Sm*Lpmo10A enhanced enzymatic saccharification of cellulose by improving the native and oxidized cello-oligosaccharides yields.

## Results and discussion

### Cloning and sequence analysis of *Sm*Bglu12A and *Sm*Lpmo10A

**Fig. 1:**
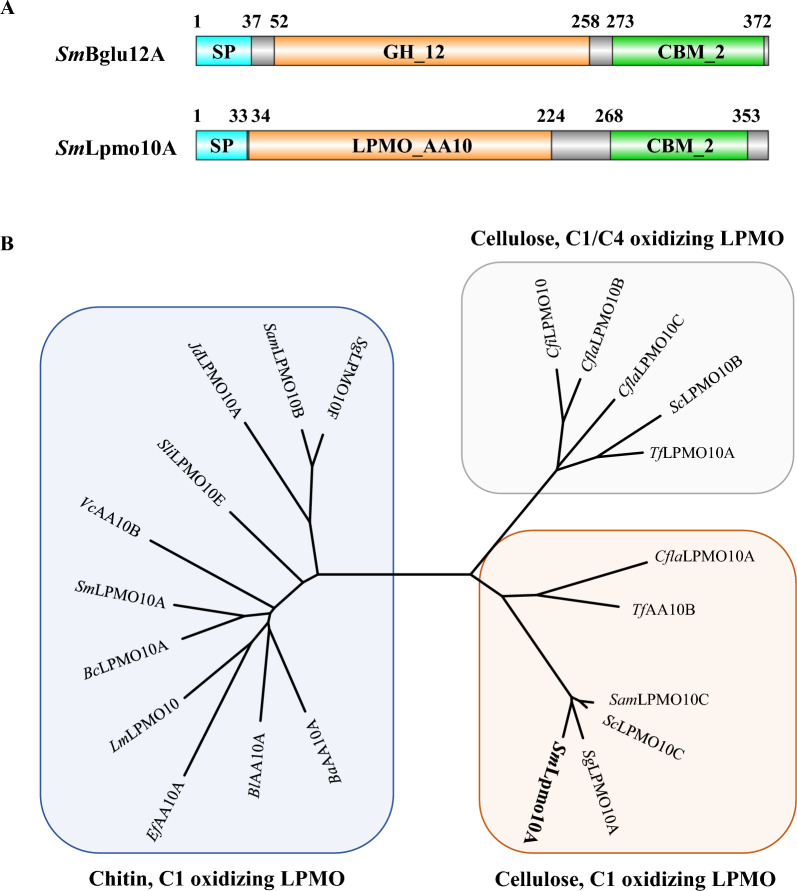
Schematic illustration of *Sm*Bglu12A and *Sm*Lpmo10A domains (**A**). SP: the signal peptide; GH_12: glycoside hydrolase family 12; CBM_2: carbohydrate binding module family 2; LPMO_AA10: lytic polysaccharide monooxygenase_auxiliary activity family 10. Evolution analysis of *Sm*Lpmo10A and characterized LPMO_AA10 (**B**). The maximum likelihood tree was constructed using MEGA7 under the default parameters. *Cfla*LPMO10A from *Cellulomonas flavigena*, D5UGB1; *Tf*AA10B from *Thermobifida fusca*, QOS58239.1; *Sam*LPMO10C from *Streptomyces ambofaciens*, A3KKC4; *Sc*LPMO10C from *Streptomyces coelicolor*, Q9RJY2; *Sg*LPMO10A from *Streptomyces griseus*, WP_164360609.1; *Cfi*LPMO10 from *Cellulomonas fimi*, F4H6A3; *Cfla*LPMO10B from *Cellulomonas flavigena*, D5UGA8; *Cfla*LPMO10C from *Cellulomonas flavigena*, D5UH31; *Sc*LPMO10B from *Streptomyces coelicolor*, Q9RJC1; *Tf*LPMO10A from *Thermobifida fusca*, Q47QG3; *Sg*LPMO10F from *Streptomyces griseus*, B1VN59; *Sam*LPMO10B from *Streptomyces ambofaciens*, WP_053126548.1; *Jd*LPMO10A from *Jonesia denitrificans*, C7R4I0; *Sli*LPMO10E from *Streptomyces lividans*, WP_003975967.1; *Vc*AA10B from *Vibrio cholerae*, Q9KLD5; *Sm*LPMO10A from *Serratia marcescens*, WP_060560026.1; *Bc*LPMO10A from *Bacillus cereus*, Q81CE4; *Lm*LPMO10 from *Listeria monocytogenes*, Q8Y4H4; *Ef*AA10A from *Enterococcus faecalis*, Q838S1; *Bl*AA10A from *Bacillus licheniformis*, Q62YN7; *Ba*AA10A from *Bacillus amyloliquefaciens*, WP_065521218.1

*Streptomyces* species have been reported to efficiently deconstruct natural polymeric macromolecules using extracellular carbohydrate-active enzymes, including cellulose, lignin, and chitin [19, 20]. Herein, two adjacent cellulolytic enzyme genes *SmBglu12A* and *SmLpmo10A* were identified from the genome of *S. megaspores* according to the annotation for carbohydrate-active enzymes against the dbCAN2 database [21]. As shown in Additional file 1, two open reading frames of *SmBglu12A* and *SmLpmo10A* were composed of 1128 bp and 1083 bp nucleotides encoding proteins of 375 and 360 amino acids, respectively. The *Sm*Bglu12A contained a signal peptide with 37 residues, a catalytic region corresponding to the family 12 of glycoside hydrolases, and a binding domain belonging to the carbohydrate binding module family 2 (Fig. 1A). The BLASTP searches against the UniProt web server showed that *Sm*Bglu12A exhibited the highest sequence identity to the exoglucanase (51.4%) from *Cellulomonas fimi*. The *Sm*Lpmo10A harbored a N-terminal signal peptide with 33 residues, a catalytic region corresponding to the auxiliary activity family 10, and a C-terminal binding domain belonging to the carbohydrate binding module family 2 (Fig. 1A). BLAST analysis showed the lytic cellulose monooxygenase from *Streptomyces ambofaciens* as the closest homolog, exhibiting 80.8% amino acid identity. Meanwhile, considering that members of the auxiliary activity family 10 of LPMOs were active on cellulose or/and chitin [6, 22], the evolutionary relationship between *Sm*Lpmo10A and characterized LPMOs in the AA10 family was further analyzed. The result indicated that *Sm*Lpmo10A might be a C1 oxidizing LPMO displayed oxidative activity on cellulose (Fig. 1B).

### Heterologous expression and purification of *Sm*Bglu12A and *Sm*Lpmo10A

*Sm*Bglu12A and *Sm*Lpmo10A were initially expressed as inclusion bodies in the *E. coli* Transetta (DE3), which might be attributed to the reducing environment of bacteria inhibiting the correct formation of disulfide bridges [23]. Based on several strategies that had been developed in recent years to improve the ability of disulfide linkages formation [24, 25], the co-expression of different chaperones plasmids in various host strains of *E. coli* was attempted to achieve soluble expression of *Sm*Bglu12A and *Sm*Lpmo10A.

**Fig. 2:**
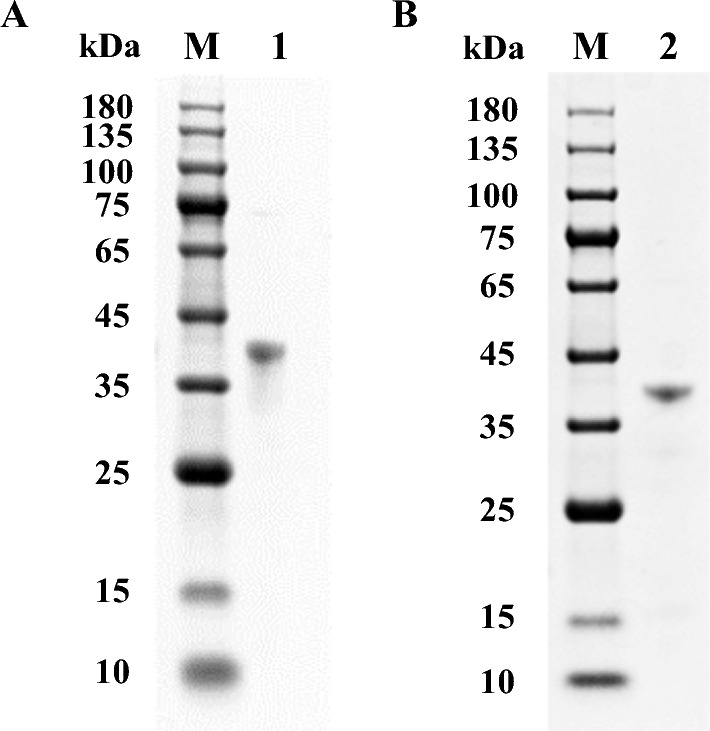
SDS-PAGE analysis of the purified *Sm*Bglu12A (**A**) and *Sm*Lpmo10A (**B**). Lanes M, 1, and 2 indicated protein marker, *Sm*Bglu12A, and *Sm*Lpmo10A, respectively

After the induction and lysis using sonication, SDS-PAGE analysis of these cell lysate supernatants showed that *Sm*Bglu12A and *Sm*Lpmo10A were successfully expressed as the soluble form in two different *E. coli* strains Origami DE3 and Shuffle T7-B with plasmids pTf16 and pG-KJE8 co-expression, respectively. The cell lysates of recombinant *Sm*Bglu12A and *Sm*Lpmo10A were applied on nickel column affinity chromatography and anion-exchange chromatography. SDS-PAGE analysis of purified recombinant *Sm*Bglu12A and *Sm*Lpmo10A showed single bands at 35–45 kDa (Fig. 2), which were similar to their calculated molecular weight of 38.7 kDa and 38.0 kDa, respectively.

### Biochemical characterization of purified *Sm*Bglu12A

**Fig. 3:**
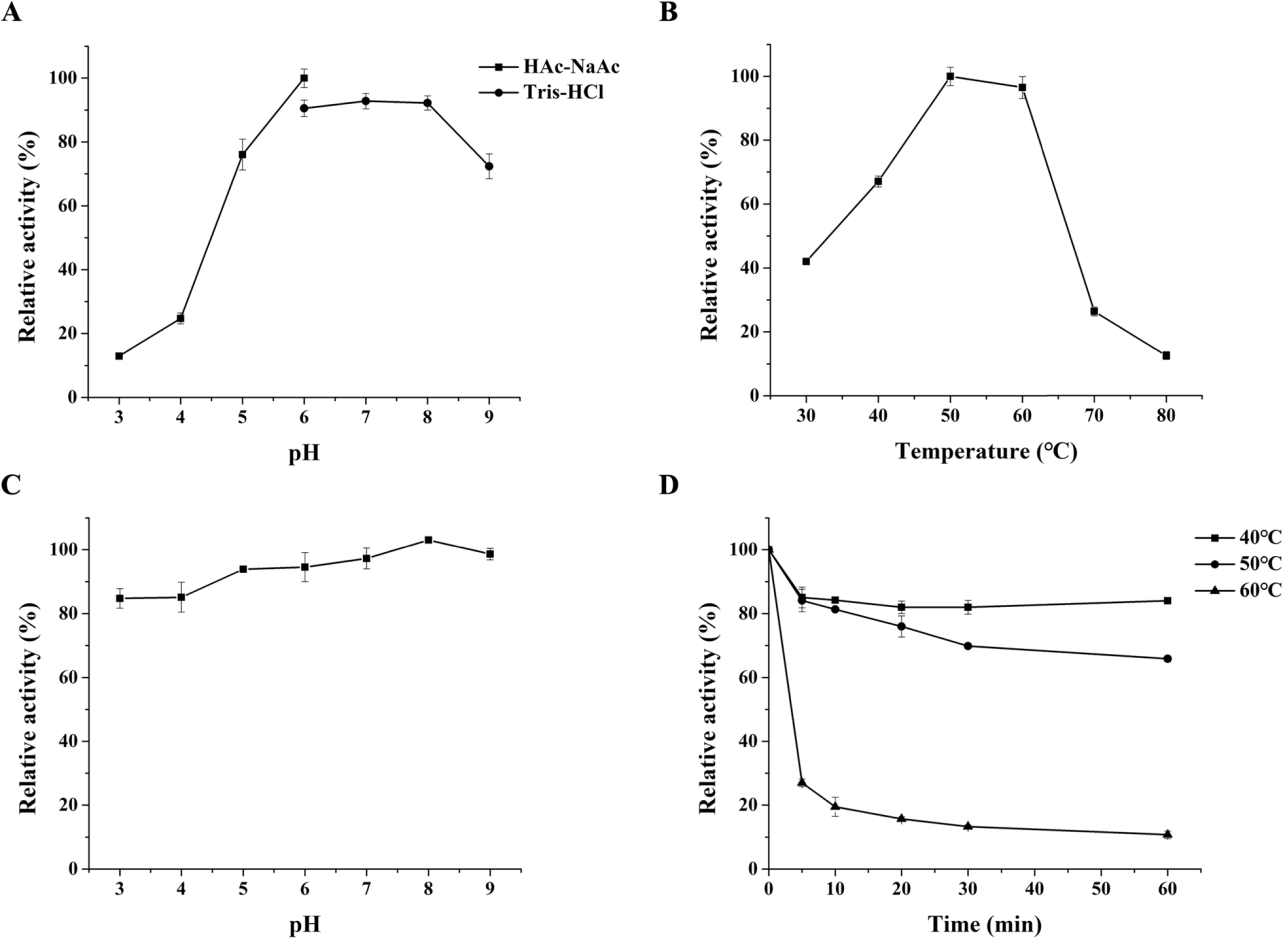
Effects of pH and temperature on the activity and stability of the purified recombinant *Sm*Bglu12A using the substrate β-1,3-1,4-glucan. **A** Optimum pH; **B** Optimum temperature; **C** pH stability. The initial activity before treatment was defined as 100%; **D** thermostability. The initial activity before treatment was defined as 100%

The purified *Sm*Bglu12A exhibited optimal pH at 6.0, with relative activities of more than 70% between pH values of 5.0 and 9.0 (Fig. 3A). In contrast, when pH was below 5.0, the enzyme activity of *Sm*Bglu12A showed a remarkable drop, exhibiting only 25% (pH 4.0) and 13% (pH 3.0) of its maximum activity. *Sm*Bglu12A was stable throughout a wide pH range of 3.0–9.0, maintaining over 80% of its initial activity after 1 h of treatment (Fig. 3C). The optimal temperature of *Sm*Bglu12A was 50 °C, and more than 95% of the maximum activity was retained at 60 °C (Fig. 3B). *Sm*Bglu12A retained 84% and 66% of its original activity when incubated at 40 °C and 50 °C for 1 h, respectively. When the temperature was raised above 60 °C, the enzyme activity of *Sm*Bglu12A decreased significantly (Fig. 3D).

The purified recombinant *Sm*Bglu12A could catalyze the hydrolysis of various substrates containing β-1,4-glycosidic bonds, including β-1,3-1,4-glucan, lichenan, carboxymethylcellulose sodium salt (CMCNa), PASC, as well as Avicel. The specific activity of *Sm*Bglu12A at pH 6.0 and 50 °C for β-1,3-1,4-glucan, lichenan, CMCNa, PASC, as well as Avicel was 239.9 ± 12.5, 189.7 ± 9.3, 49.5 ± 0.7, 43.6 ± 1.4, and 18.5 ± 4.2 U/mg (Table 1), suggesting that *Sm*Bglu12A preferentially hydrolyzed β-1,3-1,4-glucans but slightly hydrolyzed β-1,4-glucans. This feature was different from typical glucanases in GH5 or 7 families that preferentially break down β-1,4-glucans such as CMCNa [26]. Moreover, *Sm*Bglu12A was not active on laminarin (β-1,3-glucan), suggesting that it strictly hydrolyzed the β-1,4-glycosidic bonds not β-1,3-glycosidic bonds in polysaccharides. Meanwhile, the values for *K*_m_, *V*_max_, and *k*_cat_ of *Sm*Bglu12A at pH 6.0 and 50 °C were 0.3 mg/mL, 331.4 μmol/mg/min, and 201.3 s^−1^, respectively.

**Table 1:** Substrate specificities of the purified recombinant *Sm*Bglu12A for the hydrolysis of various polysaccharide substrates in the pH 6.0 Tris–HCl buffer at 50 °C

Substrate	Specific activity (U/mg)
β-1,3-1,4-Glucan	239.9 ± 12.5
Lichenan	189.7 ± 9.3
CMCNa	49.5 ± 0.7
PASC	43.6 ± 1.4
Avicel	18.5 ± 4.2

**Fig. 4:**
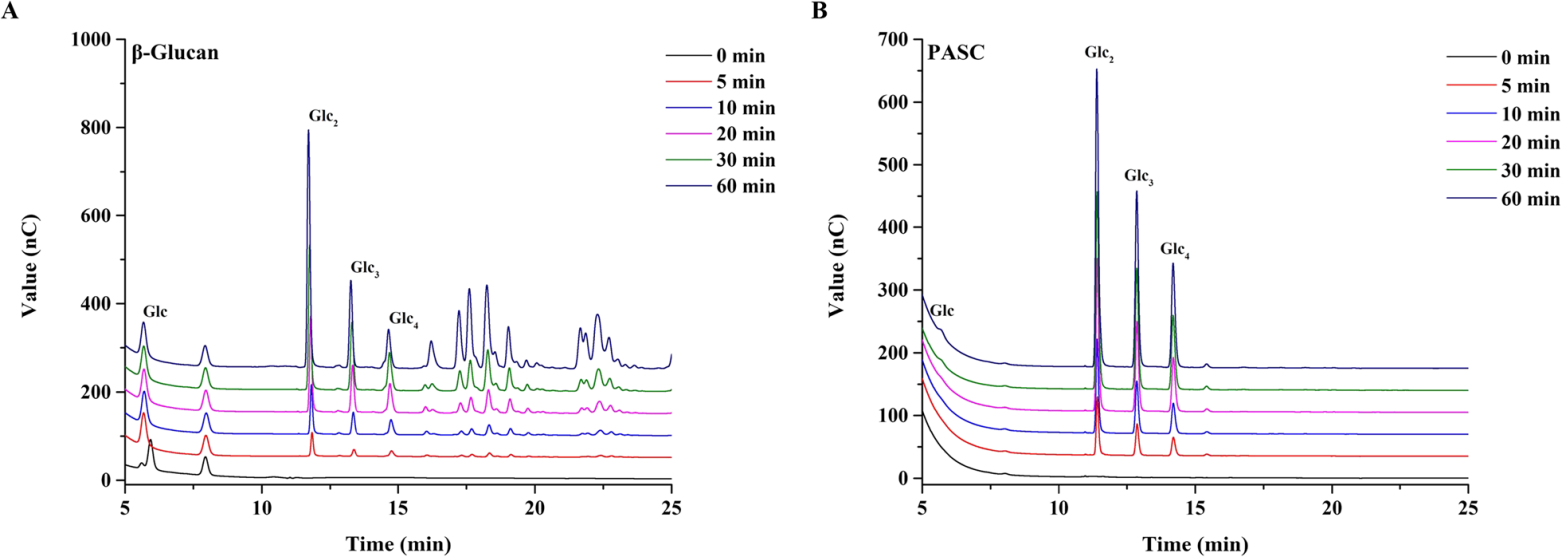
HPAEC analysis of hydrolytic products of β-1,3-1,4-glucan (**A**) and PASC (**B**) by *Sm*Bglu12A in the pH 6.0 Tris–HCl buffer at 50 °C. Glc, Glc_2_, Glc_3_, and Glc_4_ indicated glucose, cellobiose, cellotriose, and cellotetraose, respectively

To further elucidate the β-glucans degradation pattern of *Sm*Bglu12A, hydrolytic products of β-1,3-1,4-glucan and PASC were analyzed during different time periods. As shown in Fig. 4, cellobiose, cellotriose and cellotetraose were the major degradation products β-1,3-1,4-glucan and PASC at the initial stages of β-1,3-1,4-glucan and PASC hydrolysis. With the increase of reaction time, the concentration of cellobiose, cellotriose and cellotetraose increased. These results revealed that *Sm*Bglu12A was an endo-type glucanase.

### Biochemical characterization of purified *Sm*Lpmo10A

**Fig. 5:**
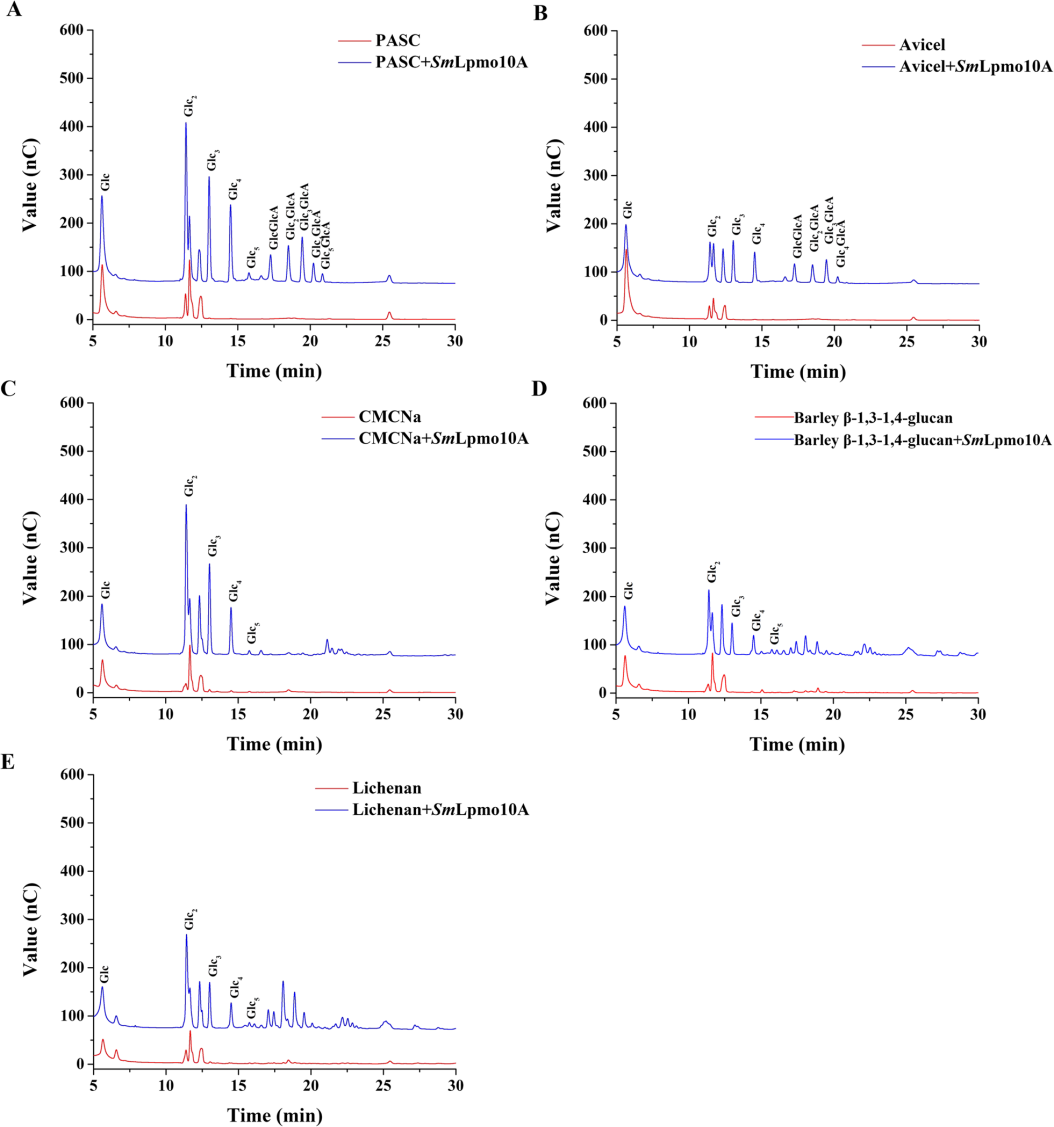
HPAEC analysis of degradation products of PASC (**A**), Avicel (**B**), CMCNa (**C**), β-1,3-1,4-glucan (**D**), and Lichenan (**E**) by *Sm*Lpmo10A in the pH 6.0 acetate buffer at 50 °C for 24 h. Glc: glucose; Glc_2_: cellobiose; Glc_3_: cellotriose; Glc_4_: cellotetraose; Glc_5_: cellopentasose; GlcGlcA: cellobionic acid; Glc_2_GlcA: cellotrionic acid; Glc_3_GlcA: cellotetraonic acid; Glc_4_GlcA: cellopentaonic acid; Glc_5_GlcA: cellohexaonic acid

The purified recombinant *Sm*Lpmo10A was incubated with PASC to assess LPMO activity and regioselectivity by analyzing the oxidized cello-oligosaccharides. As shown in Additional file 2, *Sm*Lpmo10A exhibited optimal pH at 6.0, with relative activities of more than 40% between pH values of 3.0 and 9.0. The optimal temperature of *Sm*Lpmo10A was 30 °C, and more than 60% of the maximum activity was retained at 50 °C. Besides, the purified *Sm*Lpmo10A catalyzed the oxidation of PASC to yield new chromatographic peaks (Fig. 5A), corresponding to C1 oxidized cello-oligosaccharides produced by recombinant cellobiose dehydrogenase from *Irpex lacteus* acting on cello-oligosaccharides [27]. These results indicated that the recombinant *Sm*Lpmo10A belonged to a C1-oxidizing cellulose-active LPMO.

In addition to PASC, *Sm*Lpmo10A was also found to act on various cellulosic substrates, including Avicel, CMCNa, lichenan, and β-1,3-1,4-glucan (Fig. 5). Specifically, the action of *Sm*Lpmo10A towards Avicel and PASC produced natural and oxidized cello-oligosaccharides, exhibiting a relative low specific activity on Avicel. In contrast, *Sm*Lpmo10A exhibited weak endoglucanase-like activity towards CMCNa, lichenan, and β-1,3-1,4-glucan, which was similar to the typical characteristics of AA9 LPMOs [6, 28].

### Synergistic effect between *Sm*Bglu12A and *Sm*Lpmo10A on the cellulose saccharification

**Fig. 6:**
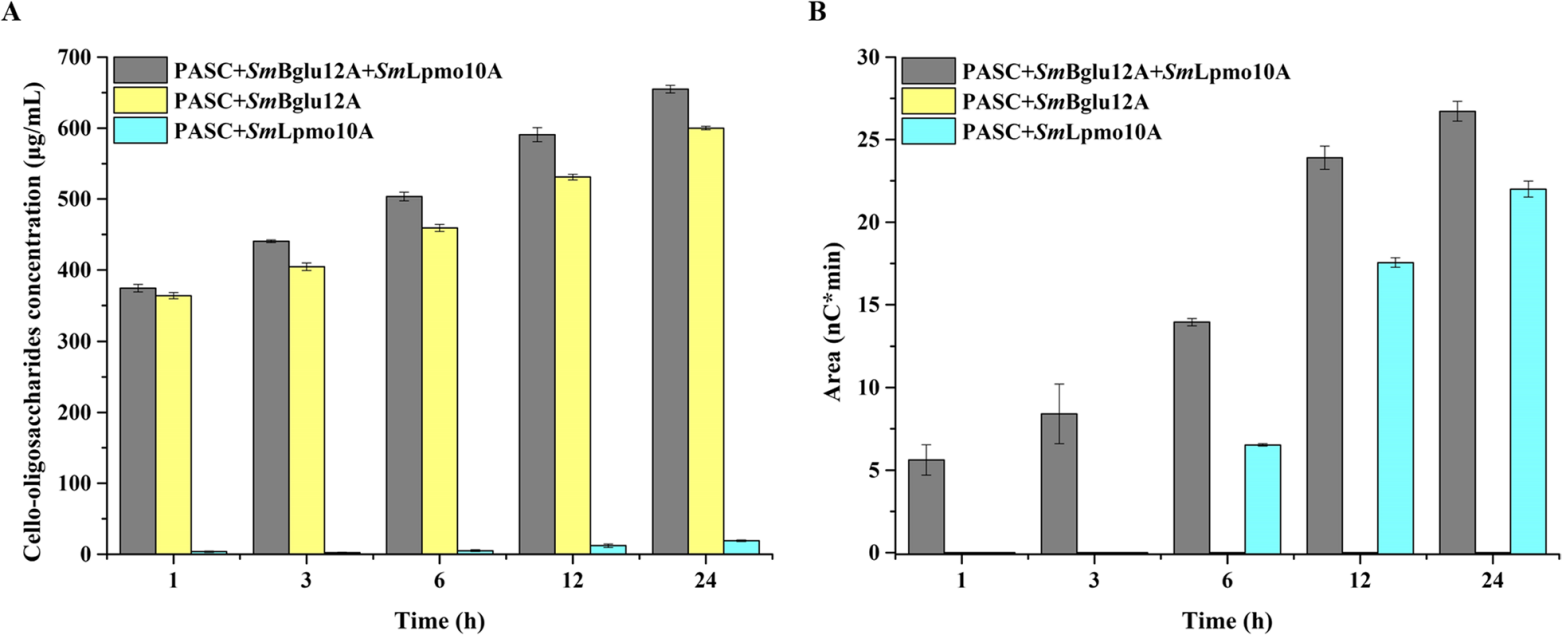
The production of native (**A**) and oxidized (**B**) cello-oligosaccharides released from PASC by *Sm*Bglu12A and *Sm*Lpmo10A in the pH 6.0 acetate buffer at 50 °C for different time periods

To evaluate whether AA10 LPMO could boost enzymatic saccharification of cellulosic substrates by GH12 β-glucanase, the synergistic effect of purified *Sm*Bglu12A and *Sm*Lpmo10A on PASC was investigated. As shown in Fig. 6A, it was observed that the production of natural cello-oligosaccharides in different groups gradually increased as time progressed. The production of natural cello-oligosaccharides obtained with the enzymes *Sm*Bglu12A and *Sm*Lpmo10A significantly exceeded the total amount produced with the sole enzyme *Sm*Bglu12A and *Sm*Lpmo10A in the whole process of saccharification, which might be attributed to more cellulose chain ends generation and crystalline cellulose surface amorphization with the action of *Sm*Lpmo10A [14]. This indicates that the presence of *Sm*Lpmo10A boosts the turnover of *Sm*Bglu12A on cellulosic substrates. After 12 h of incubation at pH 6.0 and 50 °C, the production of natural cello-oligosaccharides obtained with mixture enzymes increased by approximately 8.8% compared to the sum of natural cello-oligosaccharides produced with individual *Sm*Bglu12A and *Sm*Lpmo10A. Apart from that, there was also a significant improvement in the production of oxidized cello-oligosaccharides at the early-stage degradation (Fig. 6B). This result showed that the turnover of *Sm*Lpmo10A was also enhanced by the presence of *Sm*Bglu12A, which was similar to the phenomenon between endoglucanase (GH5 or 7) and LPMO (AA9) [7, 29]. Generally speaking, the combination of *Sm*Bglu12A and *Sm*Lpmo10A resulted in a synergistic effect on the degradation of PASC by improving the natural and oxidized cello-oligosaccharides yields.

## Conclusions

In this work, two novel cellulolytic enzyme genes *SmBglu12A* and *SmLpmo10A* from *S. megaspores* were discovered, cloned, and successfully expressed in *E. coli* strains Origami DE3 and Shuffle T7-B, respectively, after screening for multiple molecular chaperones. The recombinant *Sm*Bglu12A and *Sm*Lpmo10A are a non-typical GH12 endo-β-1,4-glucanase and a C1-oxidizing cellulose-active AA10 LPMO, respectively. Individual *Sm*Bglu12A and *Sm*Lpmo10A were both active on various cellulosic substrates, including barley β-1,3-1,4-glucan, lichenan, CMCNa, PASC, and Avicel. Most importantly, *Sm*Bglu12A and *Sm*Lpmo10A exhibited a significant synergistic activity on PASC degradation by improving the native and oxidized cello-oligosaccharides yields. This study proved for the first time that AA10 LPMO could boost the activity of GH12 glycoside hydrolases on cellulose, providing another efficient combination of glycoside hydrolase and LPMO for cellulose enzymatic saccharification.

## Methods

### Strains and plasmids

*S. megaspores* ACCC 41475 was obtained from the Agricultural Cultural Collection of China (ACCC, Beijing, China). Different *E. coli* strains containing Origami DE3 and Shuffle T7-B were purchased from ZOMANBIO Co., Ltd (Beijing, China). The expression vector pET22b and chaperone plasmids pTf16 and pG-KJE8 were purchased from Novagen Co., Ltd (Madison, USA) and Takara Bio Inc. (Beijing, China), respectively.

### Substrates and chemicals

Substrates Icelandic moss lichenan, barley β-1,3-1,4-glucan, and cello-oligosaccharides were acquired from Megazyme Ltd (Dublin, Ireland). Avicel PH-101, CMCNa, glucose, cellobiose, and beechwood xylan were obtained from Sigma–Aldrich Co., Ltd (Saint Louis, USA). Avicel PH-101 was used to prepare PASC based on the previous method reported by Zhou et al [30]. All other analytical research grade reagents and chemicals were purchased from biotech companies in China.

### Cloning, expression, and purification of *Sm*Bglu12A and *Sm*Lpmo10A

*Sm*Bglu12A and *Sm*Lpmo10A-encoding genes in the absence of signal peptide sequence were amplified from the *S. megaspores* genome with gene-specific primers (Additional file 3). Then PCR amplification products were assembled into pET22b vector digested with *Nco* I and *Not* I using the DNA assembly and cloning kit from TransGen biotech Co., Ltd (Beijing, China). After sequencing verification, the recombinant plasmids and different chaperones plasmids, including pTf16, pG-KJE8, pKJE7, pGro7, as well as pG-Tf2, were separately co-transformed into various expression hosts containing *E. coli* Origami DE3 and Shuffle T7-B for achieving soluble expression of *Sm*Bglu12A and *Sm*Lpmo10A. And the optimal combination of recombinant plasmids, chaperones plasmids, and expression hosts was selected according to the recombinant protein production through SDS-PAGE analysis.

The positive transformants of recombinant *Sm*Bglu12A and *Sm*Lpmo10A were pre-cultured in Luria broth medium at 37 °C for 12 h and used as the inoculum of 300 mL Luria broth medium supplemented with corresponding molecular chaperone inducers. *Sm*Bglu12A transformants used 0.5 mg/mL L-arabinose for inducing the expression of the molecular chaperone tig. *Sm*Lpmo10A transformants utilized 5 ng/mL tetracycline and 0.5 mg/mL L-arabinose to induce expression of multiple molecular chaperones [31]. When the cells reached exponential growth phase at OD_600_ of 0.8–1.0, a final IPTG concentration of 0.5 mM was applied.

Cells were collected after 12 h of IPTG induction at 16 °C, and sonicated at 130 W for 0.5 h. Then sonicated cell lysates were purified using nickel column affinity chromatography with 200 mM imidazole and 500 mM NaCl dissolved in the 20 mM phosphate buffer (pH 7.4). As for *Sm*Lpmo10A, the crude enzyme was further purified via HiTrap Q HP column anion exchange chromatography with a linear gradient of 0–1 M NaCl dissolved in the 20 mM phosphate buffer (pH 7.4) to remove nonspecific binding molecular chaperones. Finally, the purity of recombinant *Sm*Bglu12A and *Sm*Lpmo10A was verified by 12% SDS-PAGE. In addition, purified *Sm*Lpmo10A was saturated with CuSO_4_ for 0.5 h under room temperature using a 1:3 molar ratio, followed by removing excess copper using a PD MidiTrap desalting column with Sephadex G-25 resin from Cytiva according to the gravity protocol using the 20 mM Tris–HCl buffer (pH 7.0) [32].

### Measurement of β-glucanase and LPMO activity

β-glucanase activity was quantified by 3,5-DNS method using the substrate β-1,3-1,4-glucan [33]. The assay mixture contained 0.5% β-1,3-1,4-glucan and the diluted enzyme solution in the pH 6.0 acetate buffer. LPMO activity was evaluated through monitoring the oxidized and non-oxidized oligosaccharides released from PASC [34]. The reaction was performed at pH 6.0 acetate buffer containing 0.5% PASC and 1 mM ascorbic acid for 24 h incubation at 50 °C.

### Characterization of *Sm*Bglu12A and *Sm*Lpmo10A

The impact of pH on purified *Sm*Bglu12A was measured in the acetate buffer between 3.0 and 6.0 and Tris–HCl buffer between 6.0 and 9.0 at 50 °C. The impact of temperature on purified *Sm*Bglu12A was measured in the pH 6.0 Tris–HCl buffer ranging from 30 to 80 °C. To measure the pH stability, purified *Sm*Bglu12A was pre-incubated within the pH range from 3.0 to 9.0 at 4 °C for 1 h, and then residual activity was measured at 50 °C in the pH 6.0 Tris–HCl buffer. To determine the thermostability, purified *Sm*Bglu12A was pre-incubated at 40, 50, and 60 °C for different time periods, and then residual activity was measured at 50 °C in the pH 6.0 Tris–HCl buffer. The initial activity before treatment was defined as 100%.

The substrate specificity of *Sm*Bglu12A was studied for the hydrolysis of five different substrates in the pH 6.0 Tris–HCl buffer at 50 °C for 1 h, including β-1,3-1,4-glucan, lichenan, CMCNa, PASC, as well as Avicel. All kinetic parameters of *Sm*Bglu12A were measured using different concentrations of β-1,3-1,4-glucan varying between 1.0 and 10.0 mg/mL in the pH 6.0 Tris–HCl buffer at 50 °C for 1 h. Besides, degradation patterns of *Sm*Bglu12A on β-1,3-1,4-glucan and PASC were carried out in the pH 6.0 Tris–HCl buffer at 50 °C. High-performance anion-exchange chromatography (HPAEC) was used to monitor their corresponding degradation products.

The substrate specificity of *Sm*Lpmo10A was determined through the degradation of various cellulosic substrates, including β-1,3-1,4-glucan, lichenan, CMCNa, PASC, as well as Avicel, in the pH 6.0 acetate buffer containing 1 mM ascorbic acid at 50 °C for 24 h. The generated natural and oxidized cello-oligosaccharides were determined using HPAEC using a CarboPac PA1 analytic column (2 × 250 mm) connected to a CarboPac PA1 guard column (2 × 50 mm) at 30 °C based on the previous method reported by Westereng et al. [35]. The elution program was as follows: 0–10% B (1 M NaOAc in 0.1 M NaOH), 10 min; 10–30% B, 15 min; 30–100% B, 5 min; 100–0% B, 5 min; 100% A (0.1 M NaOH) 5 min at a flow rate of 0.25 mL/min.

### Synergistic degradation of PASC by *Sm*Bglu12A and *Sm*Lpmo10A

The synergistic action between *Sm*Bglu12A and *Sm*Lpmo10A on the enzymatic deconstruction of cellulose was evaluated in the pH 6.0 acetate buffer containing 1 mM ascorbic acid using 0.5% PASC as the substrate by addition of 0.5 μM *Sm*Bglu12A and 0.5 μM *Sm*Lpmo10A. Reactions were carried out at 50 °C for five different incubation periods (1, 3, 6, 12, and 24 h) in triplicate. The control group was set up without enzymes. The production of natural and oxidized cello-oligosaccharides from PASC was determined and quantified by HPAEC using standard mixtures of cello-oligosaccharides, including glucose, cellobiose, cellotriose, cellotetraose, cellopentaose, and cellohexose. The statistical analysis was performed using the SPSS software.

## Supplementary Information

**Additional file 1.** Nucleotide sequence analysis of two adjacent cellulolytic enzyme genes *SmBglu12A* and *SmLpmo10A* in the genome of *S. megaspores*. Genomic DNA sequences encoding *Sm*Bglu12A and *Sm*Lpmo10A were highlighted in blue and green, respectively. **Additional file 2**. Effects of pH and temperature on the activity of the purified recombinant *Sm*Lpmo10A using the substrate PASC. **A**: Optimum pH; **B**: Optimum temperature. **Additional file 3**. Primers used in this study.

## Data Availability

The datasets used and/or analyzed during the current study are available from the corresponding authors on reasonable request.

## Abbreviations

LPMOs: Lytic polysaccharide monooxygenases
AA9: Auxiliary activity family 9
PASC: Phosphoric acid swollen cellulose
CMCNa: Carboxymethylcellulose sodium salt
HPAEC: High-performance anion-exchange chromatography

## References

[1] Li T, Chen C, Brozena AH, Zhu JY, Xu L, Driemeier C, Dai J, Rojas OJ, Isogai A, Wågberg L, Hu L (2021). Developing fibrillated cellulose as a sustainable technological material. Nature.

[2] Seddiqi H, Oliaei E, Honarkar H, Jin J, Geonzon LC, Bacabac RG, Klein-Nulend J (2021). Cellulose and its derivatives: towards biomedical applications. Cellulose.

[3] Seidi F, Yazdi MK, Jouyandeh M, Habibzadeh S, Munir MT, Vahabi H, Bagheri B, Rabiee N, Zarrintaj P, Saeb MR (2022). Crystalline polysaccharides: a review. Carbohydr Polym.

[4] Singh R, Pal DB, Alkhanani MF, Almalki AH, Areeshi MY, Haque S, Srivastava N (2022). Prospects of soil microbiome application for lignocellulosic biomass degradation: an overview. Sci Total Environ.

[5] Moon M, Lee J-P, Park GW, Lee J-S, Park HJ, Min K (2022). Lytic polysaccharide monooxygenase (LPMO)-derived saccharification of lignocellulosic biomass. Bioresour Technol.

[6] Vandhana TM, Reyre J-L, Sushmaa D, Berrin J-G, Bissaro B, Madhuprakash J (2022). On the expansion of biological functions of lytic polysaccharide monooxygenases. New Phytol.

[7] Keller MB, Badino SF, Blossom BM, McBrayer B, Borch K, Westh P (2020). Promoting and impeding effects of lytic polysaccharide monooxygenases on glycoside hydrolase activity. ACS Sustain Chem Eng.

[8] Keller MB, Badino SF, Røjel N, Sørensen TH, Kari J, McBrayer B, Borch K, Blossom BM, Westh P (2021). A comparative biochemical investigation of the impeding effect of C1-oxidizing LPMOs on cellobiohydrolases. J Biol Chem.

[9] Ogunyewo OA, Randhawa A, Gupta M, Kaladhar VC, Verma PK, Yazdani SS, Kivisaar M (2020). Synergistic action of a lytic polysaccharide monooxygenase and a cellobiohydrolase from *penicillium funiculosum* in cellulose saccharification under high-level substrate loading. Appl Environ Microbiol.

[10] Tokin R, Ipsen JØ, Westh P, Johansen KS (2020). The synergy between LPMOs and cellulases in enzymatic saccharification of cellulose is both enzyme- and substrate-dependent. Biotechnol Lett.

[11] Bernardi AV, Gerolamo LE, de Gouvêa PF, Yonamine DK, Pereira LMS, de Oliveira AHC, Uyemura SA, Dinamarco TM (2021). LPMO AfAA9_B and cellobiohydrolase Afcel6A from *A. fumigatus* boost enzymatic saccharification activity of cellulase cocktail. Int J Mol Sci.

[12] Srivastava S, Jhariya U, Purohit HJ, Dafale NA (2021). Synergistic action of lytic polysaccharide monooxygenase with glycoside hydrolase for lignocellulosic waste valorization: a review. Biomass Convers Biorefin.

[13] Li Y, Li T, Guo J, Wang G, Chen G (2022). Expression and characterization of a novel lytic polysaccharide monooxygenase, *Pd*LPMO9A, from the edible fungus *Pleurotus djamor* and its synergistic interactions with cellulase in corn straw biomass saccharification. Bioresour Technol.

[14] Uchiyama T, Uchihashi T, Ishida T, Nakamura A, Vermaas JV, Crowley MF, Samejima M, Beckham GT, Igarashi K (2022). Lytic polysaccharide monooxygenase increases cellobiohydrolases activity by promoting decrystallization of cellulose surface. Sci Adv.

[15] Qin X, Zou J, Yang K, Li J, Wang X, Tu T, Wang Y, Yao B, Huang H, Luo H (2022). Deciphering the efficient cellulose degradation by the thermophilic fungus *Myceliophthora thermophila* focused on the synergistic action of glycoside hydrolases and lytic polysaccharide monooxygenases. Bioresour Technol.

[16] Zhang H, Dong S, Lou T, Wang S (2019). Complete genome sequence unveiled cellulose degradation enzymes and secondary metabolic potentials in *Streptomyces* sp. CC0208. J Basic Microbiol.

[17] Vela Gurovic MS, Díaz ML, Gallo CA, Dietrich J (2021). Phylogenomics, CAZyome and core secondary metabolome of *Streptomyces albus* species. Mol Genet Genomics.

[18] Kumar M, Kumar P, Das P, Solanki R, Kapur MK (2020). Potential applications of extracellular enzymes from *Streptomyces* spp. in various industries. Arch Microbiol.

[19] Rodríguez-Fonseca MF, Sánchez-Suárez J, Valero MF, Ruiz-Balaguera S, Díaz LE (2021). *Streptomyces* as potential synthetic polymer degraders: a systematic review. Bioengineering.

[20] Lee S, Kang M, Bae J-H, Sohn J-H, Sung BH (2019). Bacterial valorization of lignin: strains, enzymes, conversion pathways, biosensors, and perspectives. Front Bioeng Biotechnol.

[21] Zhang H, Yohe T, Huang L, Entwistle S, Wu P, Yang Z, Busk PK, Xu Y, Yin Y (2018). dbCAN2: a meta server for automated carbohydrate-active enzyme annotation. Nucl Acids Res.

[22] Guo X, An Y, Jiang L, Zhang J, Lu F, Liu F (2022). The discovery and enzymatic characterization of a novel AA10 LPMO from *Bacillus amyloliquefaciens* with dual substrate specificity. Int J Biol Macromol.

[23] Gąciarz A, Khatri NK, Velez-Suberbie ML, Saaranen MJ, Uchida Y, Keshavarz-Moore E, Ruddock LW (2017). Efficient soluble expression of disulfide bonded proteins in the cytoplasm of *Escherichia coli* in fed-batch fermentations on chemically defined minimal media. Microb Cell Fact.

[24] Rosano GL, Morales ES, Ceccarelli EA (2019). New tools for recombinant protein production in *Escherichia coli*: A 5-year update. Protein Sci.

[25] Slater SL, Mavridou DAI (2021). Harnessing the potential of bacterial oxidative folding to aid protein production. Mol Microbiol.

[26] Zhu Z, Qu J, Yu L, Jiang X, Liu G, Wang L, Qu Y, Qin Y (2019). Three glycoside hydrolase family 12 enzymes display diversity in substrate specificities and synergistic action between each other. Mol Biol Rep.

[27] Scheiblbrandner S, Ludwig R (2020). Cellobiose dehydrogenase: bioelectrochemical insights and applications. Bioelectrochemistry.

[28] Monclaro AV, Filho EXF (2017). Fungal lytic polysaccharide monooxygenases from family AA9: recent developments and application in lignocelullose breakdown. Int J Biol Macromol.

[29] Karnaouri A, Muraleedharan MN, Dimarogona M, Topakas E, Rova U, Sandgren M, Christakopoulos P (2017). Recombinant expression of thermostable processive *Mt*EG5 endoglucanase and its synergism with *Mt*LPMO from *Myceliophthora thermophila* during the hydrolysis of lignocellulosic substrates. Biotechnol Biofuels.

[30] Zhou H, Li T, Yu Z, Ju J, Zhang H, Tan H, Li K, Yin H (2019). A lytic polysaccharide monooxygenase from *Myceliophthora thermophila* and its synergism with cellobiohydrolases in cellulose hydrolysis. Int J Biol Macromol.

[31] Farajnia S, Ghorbanzadeh V, Dariushnejad H (2020). Effect of molecular chaperone on the soluble expression of recombinant fab fragment in *E. coli*. Int J Pept Res Ther.

[32] Stepnov AA, Forsberg Z, Sørlie M, Nguyen G-S, Wentzel A, Røhr ÅK, Eijsink VGH (2021). Unraveling the roles of the reductant and free copper ions in LPMO kinetics. Biotechnol Biofuels.

[33] Rykov SV, Kornberger P, Herlet J, Tsurin NV, Zorov IN, Zverlov VV, Liebl W, Schwarz WH, Yarotsky SV, Berezina OV (2019). Novel endo-(1,4)-β-glucanase Bgh12A and xyloglucanase Xgh12B from *Aspergillus cervinus* belong to GH12 subgroup I and II, respectively. Appl Microbiol Biotechnol.

[34] Villares A, Moreau C, Bennati-Granier C, Garajova S, Foucat L, Falourd X, Saake B, Berrin J-G, Cathala B (2017). Lytic polysaccharide monooxygenases disrupt the cellulose fibers structure. Sci Rep.

[35] Westereng B, Arntzen MØ, Aachmann FL, Várnai A, Eijsink VGH, Agger JW (2016). Simultaneous analysis of C1 and C4 oxidized oligosaccharides, the products of lytic polysaccharide monooxygenases acting on cellulose. J Chromatogr A.

