# Supplementary material for: Heterologous expression and characterization of novel GH12 β-glucanase and AA10 lytic polysaccharide monooxygenase from Streptomyces megaspores and their synergistic action in cellulose saccharification
Source: Biotechnol Biofuels Bioprod. 2023 May 24;16:89. doi: 10.1186/s13068-023-02332-0 (PMC10207622; doi:10.1186/s13068-023-02332-0)
Supplement: Supplementary file 1 — Additional file 1. Nucleotide sequence analysis of two adjacent cellulolytic enzyme genes SmBglu12A and SmLpmo10A in the genome of S. megaspores. Genomic DNA sequences encoding SmBglu12A and SmLpmo10A were highlighted in blue and green, respectively. Additional file 2. Effects of pH and temperature on the activity of the purified recombinant SmLpmo10A using the substrate PASC. A: Optimum pH; B: Optimum temperature. Additional file 3. Primers used in this study. [file 13068_2023_2332_MOESM1_ESM.docx]

**Additional file 1.** Nucleotide sequence analysis of two adjacent cellulolytic enzyme genes *SmBglu12A* and *SmLpmo10A* in the genome of *S. megaspores*. Genomic DNA sequences encoding *Sm*Bglu12A and *Sm*Lpmo10A were highlighted in blue and green, respectively.

cacgcagttcccctcctgtggatcacattccctgacctgcggcttcgaatttgcttcgacagtttcgtgcgcgcagccttgtcagggacatgtgagccttctacagtcccctcgaaccggctcatgggagcgctcccatcatcggggagcggaagcgctccgccggccggcccccgcaccctcttggagaggccccccac**ATGCGACGGTTACCGCACCCGATCCGCGCCGTGCGCGGTCTGGTCGCCGCCCTGCTCACCGCTCTCGCCGTGATCGCGGCGCTGGTGACCGCGACGGCACCGGCCCAGGCCGACACCACGCTCTGCGAACCGTACGGAACCACCACCATCCAGGGGCGCTACGTCGTCCAGAACAACCGCTGGGGCTCCAGCTCCCCCCAGTGCGTCACCGCCACGGACACCGGCTTCCGCCTCACGCAGGCCGACGGCTCGGTGCCGACCAACGGCGCCCCGAAGTCGTACCCGTCGGTCTTCAACGGCTGCCACTACACCAACTGCTCGCCGGGGACCAAGCTCCCGGCGCGGATCAGCGGCATCTCGAGCGCGCCCAGCAGCATCTCCTACGGCTATGTCGGCGGCGCCGTGTACAACGCCTCGTACGACATCTGGCTGGACCCGACGCCCCGGACCGACGGCGTGAACCGGACCGAGATCATGATCTGGTTCAACAAGGTGGGTCCGATCCAGCCGATCGGCTCCCAGGTCGGCACGGCCACCGTGGGCGGGCGCACCTGGCAGGTGTGGTCGGGCGGCAACGGCTCCAACGACGTGCTGTCCTTCGTCGCCCCCTCGGCGATCGAGAGCTGGAGCTTCGACGTCATGGACTTCGTCCGGGAGACCGTCGCACGCGGCATGGCGCAGAACGACTGGTACCTGACGAGTGTGCAGGCGGGCTTCGAGCCCTGGCAGAACGGTGCGGGGCTCGCGGTGAACTCCTTCTCCTCGACCGTCGACACCGGCGGCGGCACCCCGGGAGGCCCCGGCACCCCGGCGGCGTGCACGGTGTCGTACGCCACGAACGTCTGGCCGGGCGGCTTCACCGCGAACGTCACCGTGAGGAACACCGGATCGTCCGCCGTCGACGGCTGGAGGCTCGCCTTCACCCTGCCCGCCGGGCAGCGCATCACCCAAGCCTGGAACGCGTCCGTCACCCCCTCCTCGGGCACGGTCACGGCGAGCGGCCCGGACAACAACACGCGGATCGCGCCGGGCGGGAGCCAGACCTTCGGGTTCCAGGGAACCTACAGCGGCACCTTCGCGCAGCCGAACGGCTTCAGCCTGAACGGCACCGCCTGCACGACCGCGTGA**cgcgaccgcgggccgcgggccggccactcggccccctcaccaccccccagtccctcgccacccccagtcccctcgcccgcccggtcgatgcccaccccgggcgggcgagggttccagctctttctccaagtcatgatcggttcactgtcgggacgggagatccc**ATGACCTTACGCAGCAGATTCGTCTCCTTGGCGGCGGTACTGGCCACCCTGCTCGGAGGGCTCGGCCTGAGCTTCCTCTGGCAGAACAACGCGCAGGCGCACGGTGTGGCGATGGTGCCCGGCTCGCGCACCTACCTCTGCCAGCTGGACGCCATCACCGGCACCGGCGCGCTGAACCCGACCAACCCGGCGTGCCGGGACGCGCTGAACAAGAGCGGCTCGTCGGCGCTGTACAACTGGTTCGCCGTCCTCGACTCCCGGGCCGCCGGACGGGGCCCCGGCTACGTGCCGGACGGCACGCTCTGCAGCGCCGGTGACCGGTCCCCGTACGACTTCTCCGCCTACAACGCGGCCCGCGCCGACTGGCCCCGGACGCATCTGACGTCCGGAGCGACGGTGAAGGTGCAGTACAGCAACTGGGCGGCCCACCCCGGCGACTTCCGGGTCTACCTCACCAAGCCCGGCTGGTCGCCCACCTCCCCGCTGGGCTGGAACGACCTGGAGCTCATCCAGACCGTCACCAACCCGCCCCAGCAGGGTTCGCCGGGCACCAACGGCGGCCACTACTACTGGGACCTGAAGCTGCCCTCCGGACGCTCCGGCGACGCGCTGATCTTCATGCAGTGGGTCCGTTCGGACAGCCAGGAGAACTTCTTCTCCTGCTCCGACATCGTCTTCGACGGCGGCAACGGCGAGGTGACCGGCATCCGGAACCCGGGCGGTACGCCGACCCCGACCCCGACCCCGACGCCGCCCACCACACCGCCGCACACCGGTTCCTGCATGGCCGTCTACAACGTGGTGAACTCCTGGAACGGTGGCTTCCAGGGCTCCGTCGAGGTGATGAACCACGGGACGTCGCCGCTCAGCGGCTGGGCCGTGCGCTGGCAGCCCGGTTCCGGGACCCGGATCAGCAGTGTGTGGAACGGGTCCCTGTCCACCGGCTCCGACGGAGCGGTGACGGTGAGGAACGTCGATCACAACCGTGTCGTCAACCCCGACGGGAGCGTGACGTTCGGCTTCACGGCCACCTCGTCGGGCAACGACTTCCCGACGGGAACGATCGGCTGCGTGGAGCCGTAG**cgtccgacccgtcgaccggaggcgccgcgccacggcggcgtacgggcacgacggccgggaggcagggccgggttcccccgtggaacccggccctgcgcccgcccgccgctgcgacctcggggcggacggcggggccgtcgcctcctggaccgcgaccggtagtgcgtcgccggt

**Additional file 2.** Effects of pH and temperature on the activity of the purified recombinant *Sm*Lpmo10A using the substrate PASC. A: Optimum pH; B: Optimum temperature.


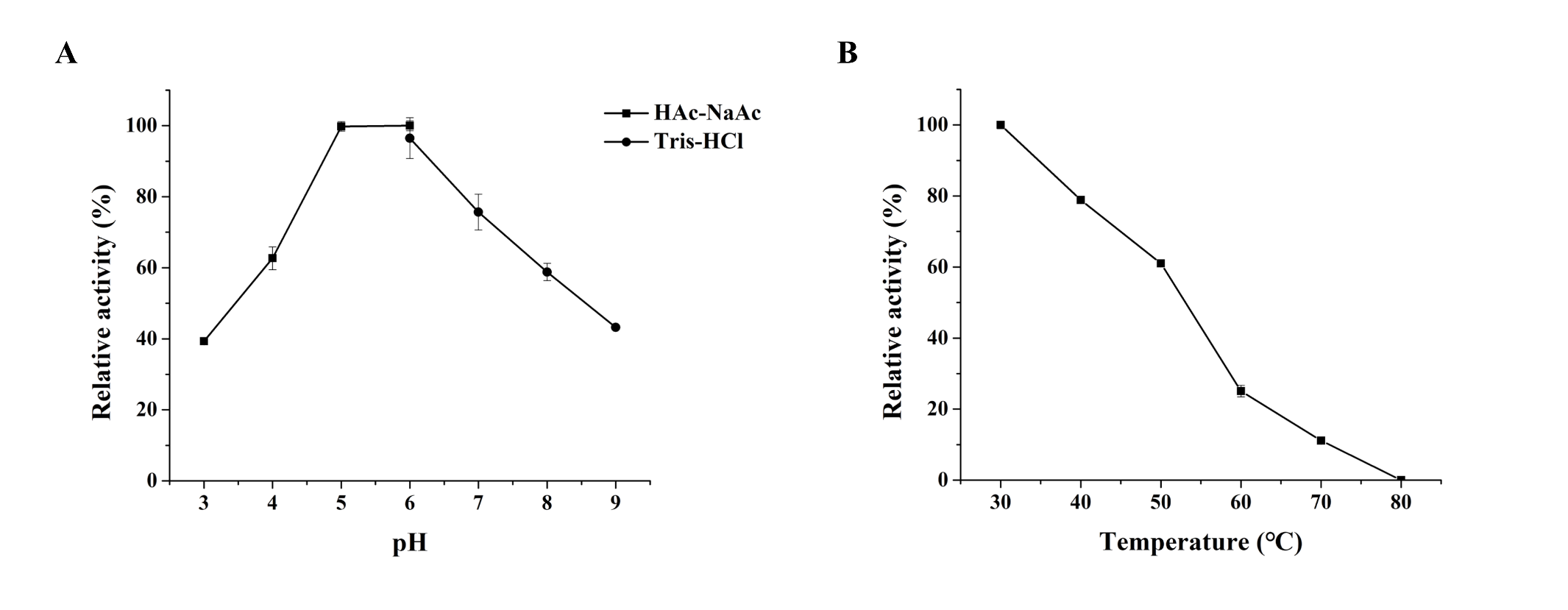


**Additional file 3.** Primers used in this study.

| **Primer** | **Nucleotide sequence (5′→3′)^a^** |
| --- | --- |
| *Sm*Bglu12A-*Nco* I-F | ***CCTCGCTGCCCAGCCGGCGATGGCC***GACACCACGCTCTGCGAACCGTAC |
| *Sm*Bglu12A-*Not* I-R | ***GGTGGTGGTGCTCGAGTGCGGCCGC***CGCGGTCGTGCAGGCGGTGC |
| *Sm*Lpmo10A-*Nco* I-F | ***CCTCGCTGCCCAGCCGGCGATGGCC***CACGGTGTGGCGATGGTGCCCGGC |
| *Sm*Lpmo10A-*Not* I-R | ***GGTGGTGGTGCTCGAGTGCGGCCGC***CGGCTCCACGCAGCCGATCGTTCCC |

^a^The homologous sequences are highlighted in bold and italic.
